# 3D Imaging through Scatterers with Interferenceless Optical System

**DOI:** 10.1038/s41598-018-19344-8

**Published:** 2018-01-18

**Authors:** Saswata Mukherjee, A. Vijayakumar, Manoj Kumar, Joseph Rosen

**Affiliations:** Department of Electrical and Computer Engineering, Ben-Gurion University of the Negev, P.O. Box 653, Beer-Sheva, 8410501 Israel

## Abstract

Imaging through a scattering medium is a challenging task. We propose and demonstrate an interferenceless incoherent opto-digital technique for 3D imaging through a scatterer with a single lens and a digital camera. The light diffracted from a point object is modulated by a scattering mask. The modulated wavefront is projected on an image sensor using a spherical lens and the impulse response is recorded. An object is placed at the same axial location as the point object and another intensity pattern is recorded with identical experimental conditions and with the same scattering mask. The image of the object is reconstructed by a cross-correlation between a reconstructing function and the object hologram. For 3D imaging, a library of reconstructing functions are created corresponding to different axial locations. The different planes of the object are reconstructed by a cross-correlation of the object hologram with the corresponding reconstructing functions.

## Introduction

Light is scattered when it encounters an optically inhomogeneous media and it is often a difficult task to image objects through scattering media^1–3^. However, this fundamental problem needs to be addressed in order to image through naturally occurring inhomogeneous scatterers such as biological tissues^4^, fog^5^, and other turbid media^6^. Various coherent digital holography techniques have been developed to image through a scattering medium^7–9^. While different techniques are being developed to nullify or minimize the effects of a scatterer in order to image objects through it, in other studies, scattering has also been used as a tool to improve the characteristics of imaging. For instance, a scattering medium has been employed to improve the lateral resolution of imaging^10,11^. Recently, digital scattering masks with controllable scattering ranks have been used as tools to improve axial^12^ and lateral resolution^13^ in imaging applications.

Laser-based imaging techniques have been the first choice for imaging through scattering medium owing to their high optical power^7–9,11^. However, the inclusion of laser into the optics configuration results in a high cost and creates many undesirable imaging effects including edge ringing and speckle. In References^12,13^ an incoherent source of light is used which reduces the cost of the imaging system, makes it eye safe and also improves the lateral resolution. However, the use of one and two spatial light modulators (SLMs) respectively, increases the cost of the optical configuration. A simple, lensless, low-cost incoherent imaging system has been proposed and demonstrated for imaging objects through a scattering medium^14^. However, the technique is limited to 2D imaging and the reconstruction of the image involves the use of a time-consuming iterative Fienup type algorithm^15^. Similarly, another incoherent super-resolution microscopy technique using a scattering mask^16^ has also been shown, requiring an iterative and also time-consuming Richardson–Lucy deconvolution algorithm^17^ for the reconstruction of the object and lacking 3D imaging capability. A lensless 3D imager^18^ using incoherent light based on compressive sensing has been recently shown.

Wavefront shaping techniques with dynamic feedback have been developed to image objects through turbid media. However, these techniques involve time-consuming dynamic corrections with complicated algorithms and also requires an SLM^19,20^. There are numerous imaging techniques to image objects through a scattering medium such as Monte Carlo analysis in two-photon fluorescence^21^, confocal imaging with an annular pupil^22^, absorption study^23^, etc. Another coherent digital holography technique has been demonstrated for 3D imaging and phase retrieval but the optical configuration is complex with many optical components^24^.

An incoherent digital holography based adaptive optics technique^25,26^ was demonstrated based on Fresnel incoherent correlation holography technique^27^ to image through turbid media but the technique requires an interferometer to record a guide star and object holograms. Another lensless incoherent 3D imaging^28^ technique for retrieving objects embedded between dynamic scatterers has been proposed and experimentally demonstrated, but it requires a reference point at an off-axis point for calibration purpose, thus making the setup difficult to align. The use of a reference point together with an object induces certain limitations on the setup such as a limited field of view. Recently, a scatter-plate microscope^29^ which uses the scatterer as a tunable objective lens of the microscope has been demonstrated which can detect objects through scattering layers, but the reconstruction procedure has not shown 3D imaging capability. Similarly, another incoherent imaging technique^30^ was recently demonstrated which utilizes a known reference object to reconstruct the object of interest, but 3D imaging was not shown. A phase-diversity non-invasive speckle imaging method^31^ was reported which can image through a thin scatterer. However, the method requires multiple camera shots from different positions of the sensor and 3D imaging is not demonstrated.

In this study, we propose an interferenceless, motionless, incoherent digital holography technique without any SLM and laser for imaging objects through scattering sheet. Unlike the above-mentioned techniques, the method does not involve any iterative algorithm^14–17^, dynamic corrections^19,20^, and complicated experimental setup^21–26^. For 2D imaging, the technique enables to observe the hidden object through the scatterer. For 3D imaging, the scatterer becomes the tool which enables 3D imaging by a single camera shot, or by two shots when higher SNR is desired.

## Methodology

**Figure 1:**
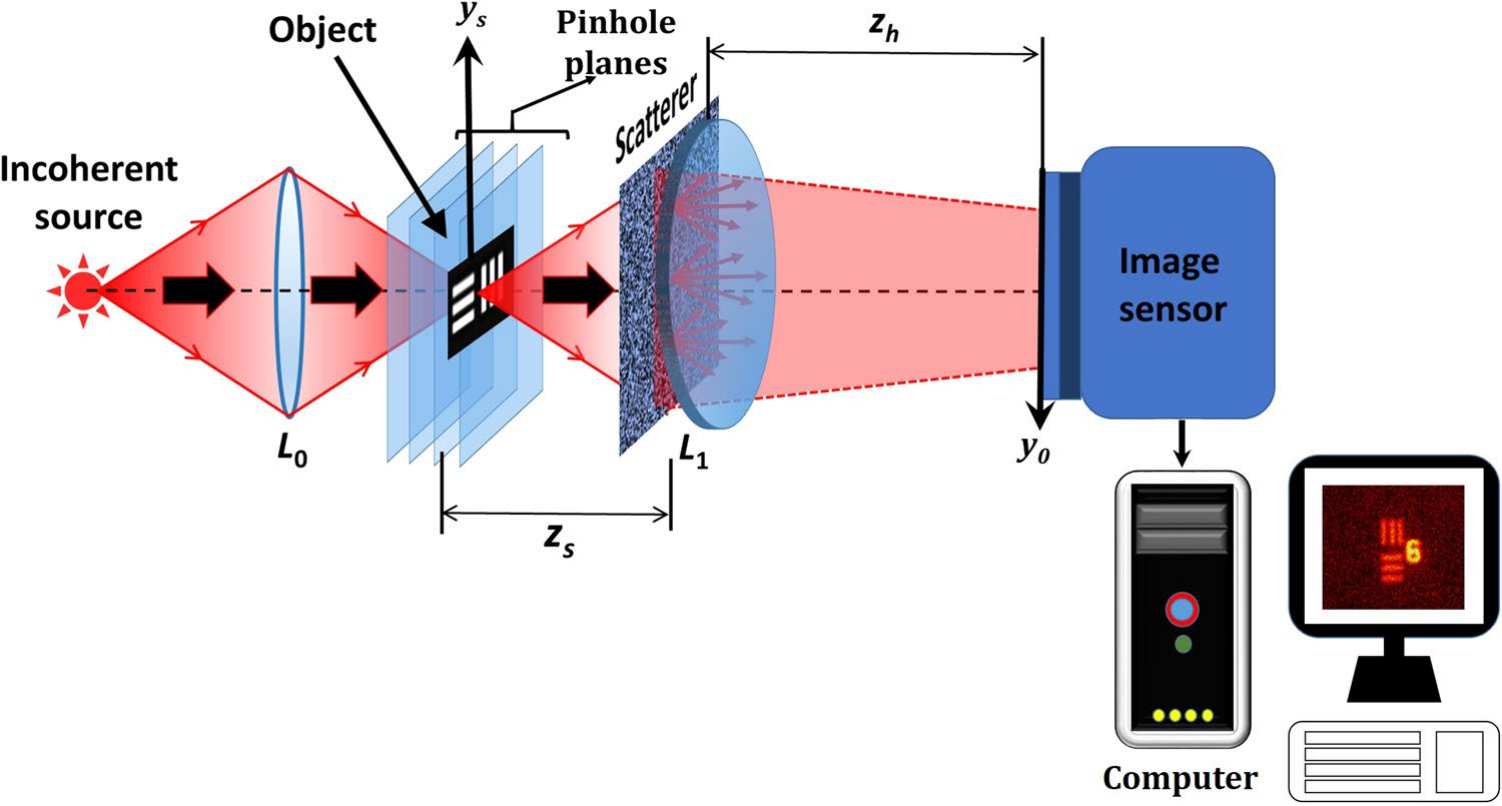
Optical configuration of 3D imaging system with scatterer.

The optical configuration of the proposed technique is shown in Fig. 1. Light from an incoherent source is used to critically illuminate a point object using a lens *L*_0_. The light diffracted from the point object is diffused by a thin scattering sheet located at a distance of *z*_*s*_ from the object. The diffused light is collected and focused by a refractive lens *L*_1_ with a focal length *f*_1_ = (1/*z*_*s*_ + 1/*z*_*h*_)^−1^, where, *z*_*h*_ is the distance between *L*_1_ and the image sensor. In the absence of the scatterer, a focused image of the point object is obtained on the sensor plane. Hence, the role of *L*_1_ is to project the entire optical energy into the camera aperture, and by that to maintain the optical efficiency optimally. It is well-known that a positive lens illuminated by a quasi-monochromatic point source yields, at the image plane of the point source, a 2D Fourier transform (multiplied by a quadratic phase function which is vanished by recording the intensity) of any aperture positioned between the source and the image point^32^. The center of the Fourier transform coincides with the image point. Therefore, if the source point is at ${\bar{r}}_{s}$, the intensity on the sensor plane is located at ${\bar{r}}_{s}{z}_{h}/{z}_{s}$.

For an object point at ${\bar{r}}_{s}$, the intensity pattern on the image sensor is given by,

1 $$\begin{array}{rcl}{I}_{PSH}({\bar{r}}_{0}) & = & C{|\nu [\frac{1}{\lambda {z}_{h}}]{\Im\!\!\!\mbox{---}} [L(\frac{{\bar{r}}_{s}}{{z}_{s}})\exp ({i{\rm{\Phi }}}_{{\rm{r}}})]|}^{2}\\  & = & C{|\nu [\frac{1}{\lambda {z}_{h}}]{\Im\!\!\!\mbox{---}} [\exp ({i{\rm{\Phi }}}_{{\rm{r}}})]|}^{2}\ast \delta ({\bar{r}}_{0}-\frac{{z}_{h}}{{z}_{s}}{\bar{r}}_{s})\end{array}$$

where ν is the scaling operator such that *ν*[*α*]*f*(*x*) = *f*(*αx*), *λ* is the wavelength, the star * stands for 2D convolution, ${\Im\!\!\!\mbox{---}}$ indicates 2D Fourier transform, *C* is a constant, ${\bar{r}}_{0}=({x}_{0},{y}_{0})$ is the transverse location vector on the sensor plane, *L* represents linear function, given by, $L(\bar{s}/z)=\exp [i2\pi {(\lambda z)}^{-1}({s}_{x}x+{s}_{y}y)]$ and Φ_*r*_ is the caustic phase profile of the scatterer. The intensity *I*_*PSH*_ is the impulse response of the system termed as the point spread hologram (PSH). For 3D imaging, the location of the point object is shifted to different axial locations and with the same scatterer, a library of PSHs is created.

The intensity of a general 2D object can be considered as a collection of shifted independent point sources and can be expressed as

2 $$o({\bar{r}}_{s})=\sum _{j}^{N}{a}_{j}\delta ({\bar{r}}_{s}-{\bar{r}}_{j}),$$

where each *a*_*j*_ is a positive real constant. The multi-points 2D object is placed at the same axial location as the point object and an intensity pattern is recorded through the same scatterer. Since the system from the input object to the camera plane is linear and space invariant, the intensity of the image sensor is given by

3 $${I}_{OBJ}({\bar{r}}_{0})=\sum _{j}{a}_{j}{I}_{PSH}({\bar{r}}_{0}-\frac{{z}_{h}}{{z}_{s}}{\bar{r}}_{j}).$$

In other words, the intensity on the camera plane is the convolution between the object *O* and the PSH, i.e. ${I}_{OBJ}=O\ast {I}_{PSH}$. The goal is to reconstruct the object *O* from the camera intensity *I*_*OBJ*_. To figure out the best way to reconstruct the object, we consider the Fourier transform of the camera intensity as the following,

4 $${\tilde{I}}_{OBJ}=\,{\Im\!\!\!\mbox{---}} \,\,\{O\ast {I}_{PSH}\}=\tilde{O}\cdot |{\tilde{I}}_{PSH}|\exp (i\,\text{arg}\{{\tilde{I}}_{PSH}\}),$$

where, ${\tilde{I}}_{OBJ},\tilde{O}\,$ and $\,{\tilde{I}}_{PSH}$ are 2D Fourier transforms of ${I}_{OBJ},O\,{\rm{and}}\,{I}_{PSH}$, respectively. Apparently, to reconstruct the object from ${\tilde{I}}_{OBJ}$ of Eq. (4), one needs to multiply ${\tilde{I}}_{OBJ}$ with the inverse filter ${|{\tilde{I}}_{PSH}|}^{-1}\exp (-i\,\text{arg}\{{\tilde{I}}_{PSH}\})$ and inversely Fourier transform the product. However, such operation gains the noise on the spectral plane to an unaccepted level. Hence, a more practical strategy is to multiply ${\tilde{I}}_{OBJ}$ with the filter ${|{\mathop{I}\limits^{ \sim }}_{PSH}|}^{\gamma }\exp (-i\,\text{arg}\{{\mathop{I}\limits^{ \sim }}_{PSH}\})$, where γ is a real number chosen as the number that yields minimum error between the reconstructed and the ideal objects. Using such filter, the images are reconstructed with a transverse magnification *M*_*T*_ = (*z*_*h*_/*z*_*s*_). The reconstructed image can be expressed as,

5 $$\begin{array}{rcl}P({\bar{r}}_{R}) & = & {\Im\!\!\!\mbox{---}}^{-1}\{{\tilde{I}}_{OBJ}{|{I}_{PSH}|}^{\gamma }\exp (-i\,\text{arg}\{{\tilde{I}}_{PSH}\})\}\\  & = & \sum _{j}{a}_{j}{\rm{\Lambda }}({\bar{r}}_{R}-\frac{{z}_{h}}{{z}_{s}}{\bar{r}}_{j})\approx o(\frac{{\bar{r}}_{s}}{{M}_{T}})\end{array}$$

where Λ is a delta-like function and ${\bar{r}}_{R}=({x}_{R},{y}_{R})$ is the transverse location vector on the reconstruction plane.

The reconstructed image is usually accompanied by a relatively strong background noise^12,13^. The SNR can be improved by synthesizing a bipolar hologram for both, the point and the object, by recording two intensity patterns using two different scattering sheets. The first intensity patterns *I*_*PSH1*_ and *I*_*OBJ1*_ are recorded using a first scattering sheet. Similarly, *I*_*PSH2*_ and *I*_*OBJ2*_ are recorded using a different independent second scattering sheet. The bipolar holograms for the point object (*H*_*PSH*_ = *I*_*PSH1*_ − *I*_*PSH2*_) and for the object (*H*_*OBJ*_ = *I*_*OBJ1*_ − *I*_*OBJ2*_) are synthesized by subtracting their respective intensity patterns. Consequently, in terms of the spatial spectrum of Eq. (4), the zeroth order of the spectrum is minimized by synthesizing the bipolar holograms, which makes the magnitude of $\,{\tilde{I}}_{PSH}$ and ${\tilde{I}}_{OBJ}$ more uniform than the case of the single camera shot. Thus, each reconstructed point becomes sharper with less background noise. The SNR can be further improved based on the assumption that any two different scatterers have a negligible cross-correlation value compared to their autocorrelation value and so by averaging the complex reconstructions using several sets of scatterer pairs, can reduce the background noise further.

**Figure 2:**
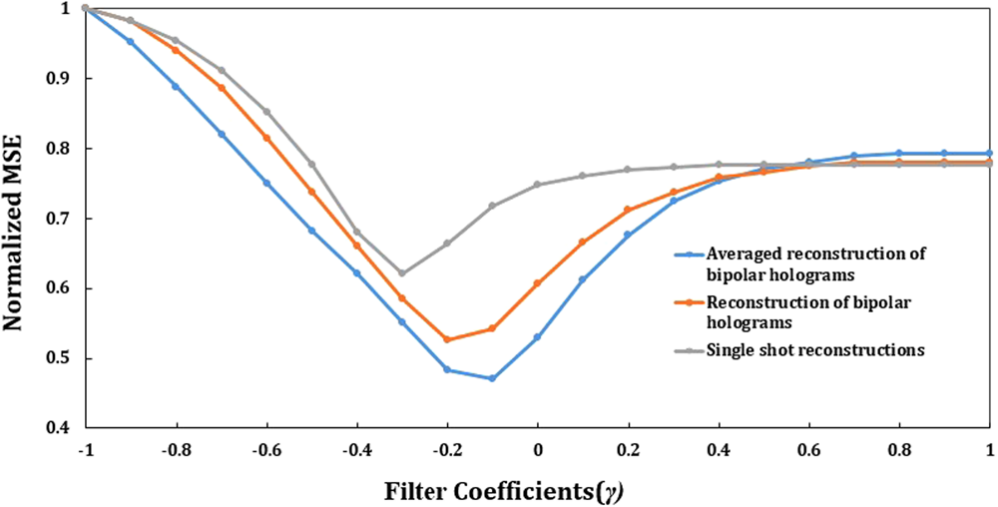
Plot of normalized mean square error (MSE) over filter coefficients *γ*.

The optimal value of *γ* was searched in the ranges from −1 to 1, with *γ* = −1 corresponding to an inverse filter, *γ* = 0 representing a phase-only filter and *γ* = 1 is a matched filter, accordingly. Along this range, we calculated the mean square error (MSE) given by,

6 $$MSE=\frac{1}{M\cdot N}{\sum _{M}\sum _{N}|{\hat{O}}_{mn}-\mu {O}_{mn}|}^{2}$$

where $\hat{O}$ denotes the image matrix on the image sensor of the system of Fig. 1, without the scatterer, and *O* is the reconstructed image with the scatterer. *M* and *N* are the number of rows and columns of the matrices, respectively. The reconstructed object has been normalized by a constant *μ*^33,34^. As is shown in Fig. 2 the minimum MSE is obtained in the case of averaging over 3 sets of scatterer pairs, for *γ* = −0.1. Note, that although a single bipolar hologram yields a minimal MSE for *γ* = −0.2, the minimum MSE of the average reconstructions is obtained for *γ* = −0.1, in each bipolar hologram.

### Experiments

**Figure 3:**
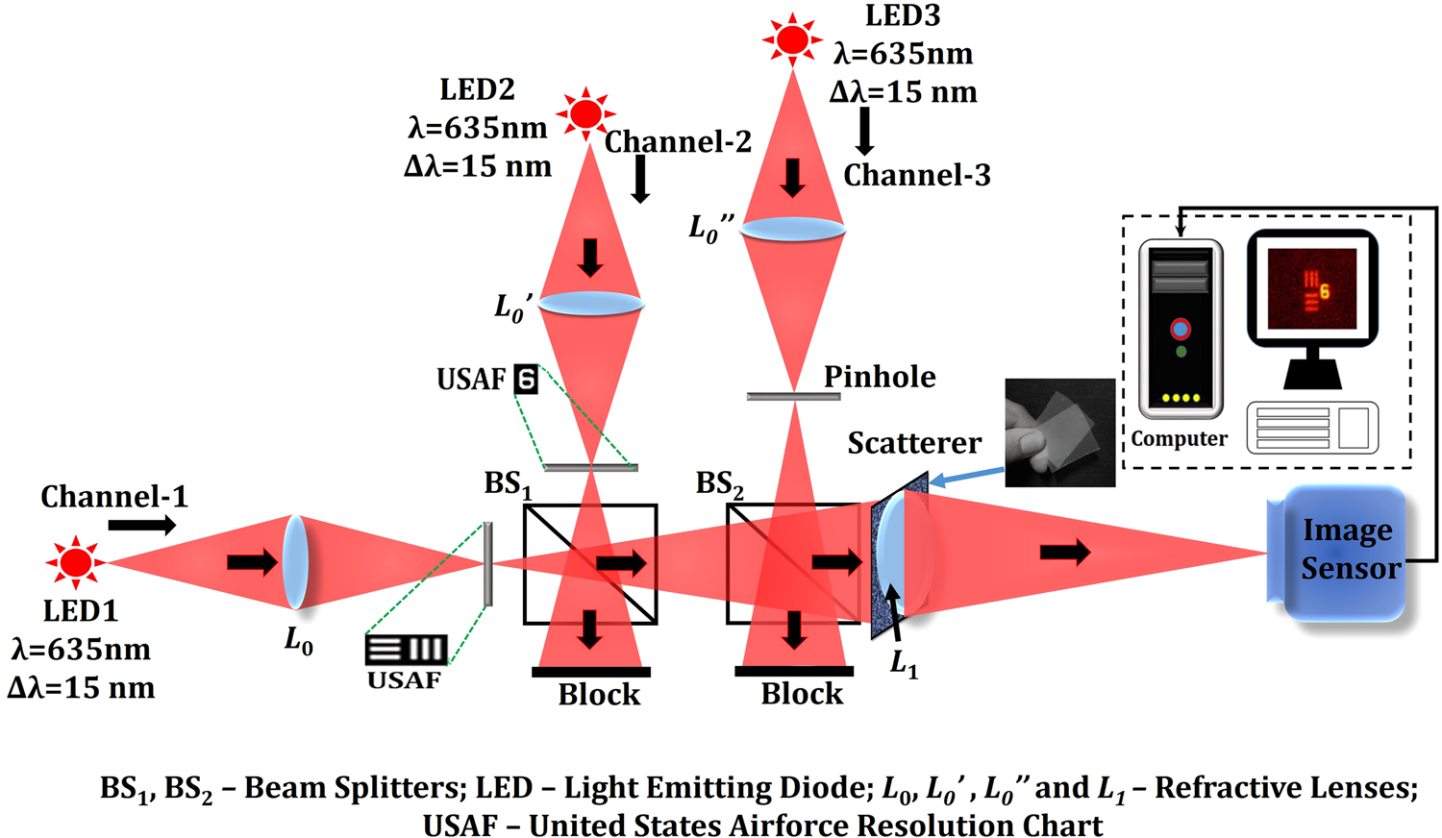
Experimental setup with a scatterer.

The schematic of the experimental setup is shown in Fig. 3. The setup consists of three illumination channels with identical high power LEDs (Thorlabs LED635L, 170 mW, λ = 635 nm, Δλ = 15 nm) emitting light at λ = 635 nm. Three channels have been adjusted such that a pinhole and two objects can be illuminated simultaneously so that the accurate axial locations of the pinhole and the objects can be validated. Three identical refractive lenses *L*_0_, *L*_0_’ and *L*_0_” were used to critically illuminate three objects: (1) element 6 of group 2 in the United States Air Force (USAF) resolution chart with 7.13 lp/mm, (2) numeric digit 6 corresponding to the element 6 of group 2 in the United States Air Force (USAF) resolution chart, and (3) a pinhole with a diameter of approximately 100 μm. In the single plane experiment, the distance between the pinhole and the scatterer (*z*_*s*_) is *z*_*s*_ = 11.7 cm. The distance from lens *L*_1_ with a focal length *f*_1_ = 5 cm to the image sensor (GigE vision GT Prosilica, 2750 × 2200 pixels, 4.54 μm pixel pitch) is 9 cm calculated from the equation *z*_*h*_ = *z*_*s*_*f*_1_/(*z*_*s*_ − *f*_1_). The diameter of the lens *L*_1_ is 5 cm imposing a numerical aperture (NA) of approximately 0.2 (*D*/2*z*_*s*_). The corresponding lateral resolution is ≈ 2 μm (0.61λ/NA). However, the larger diameter of the pinhole prevents achieving the highest possible lateral resolution as allowed by the NA. As a consequence, the lateral resolution of the optical system is dictated by the diameter of the pinhole. A pinhole with a smaller diameter can yield a higher lateral resolution but might decrease the light power and subsequently the signal to noise ratio (SNR). In the present setup, due to the power constraints of the given LED, it is impossible to use a smaller pinhole. The scatterer is a low-cost scattering sheet shown as an inset in Fig. 3. It can be noted that, in the absence of the scatterer, the optical setup is a three channel, single lens imaging system. The pinhole and the two objects in the three channels are aligned in the absence of the scatterer such that the pinhole is in between the center of the two objects, and also at the center of the image sensor.

## Experimental Results

**Figure 4:**
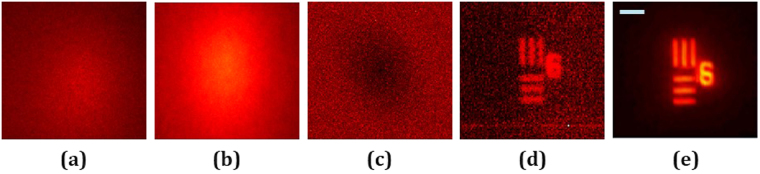
Intensity patterns at the image sensor of (**a**) *I*_*PSF*_, for the pinhole in the input, and (**b**) *I*_*OBJ*_, for objects in the input. (**c**) Image of the filter magnitude with *γ* = −0.3. (**d**) Single shot reconstruction of objects at the same plane with a filter of *γ* = −0.3. (**e**) Regular imaging without any scattering medium. Scale bar: 350 μm.

The experiment is carried out by attaching the scatterer close to the lens *L*_1_ and recording the intensity patterns for the pinhole *I*_*PS*F_ and for the USAF objects *I*_*OBJ*_ by blocking the other channels, respectively. The images of the *I*_*PSF*_ and *I*_*OBJ*_ are shown in Fig. 4(a) and (b), respectively. Note that the image in Fig. 4(b) is actually the image of the object through the scattering sheet without any processing. Needless to say, the object cannot be directly recognized by this image. The reconstructing filter is given as ${|{\mathop{I}\limits^{ \sim }}_{PSH}|}^{\gamma }\exp (-i\,\text{arg}\{{\mathop{I}\limits^{ \sim }}_{PSH}\})$ where the optimal *γ* according to Fig. 2 is *γ* = −0.3. The image of the filter is shown in Fig. 4(c). The reconstruction results when the two objects are located at the same distance is shown in Fig. 4(d). The regular imaging of the two objects free of any scattering medium is shown in Fig. 4(e).

**Figure 5:**
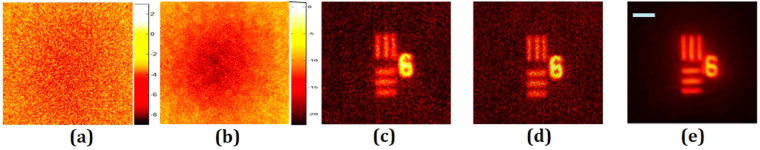
Bipolar holograms of (**a**) pinhole (*H*_*PSH*_) and (**b**) objects (*H*_*OBJ*_) in the system input, (**c**) reconstructed image by a single bipolar hologram with *γ* = −0.2, (**d**) averaged image of complex reconstructions of bipolar holograms, and (**e**) regular imaging of the two objects without any scattering medium. Scale bar: 350 μm.

The bipolar holograms of the pinhole *H*_*PSH*_ and object *H*_*OBJ*_ are shown in Fig. 5(a) and (b), respectively. The reconstructed images without and with averaging are shown in Fig. 5(c) and (d), respectively, obtained with the filter parameters *γ* = −0.2 and *γ* = −0.1, respectively.

**Figure 6:**
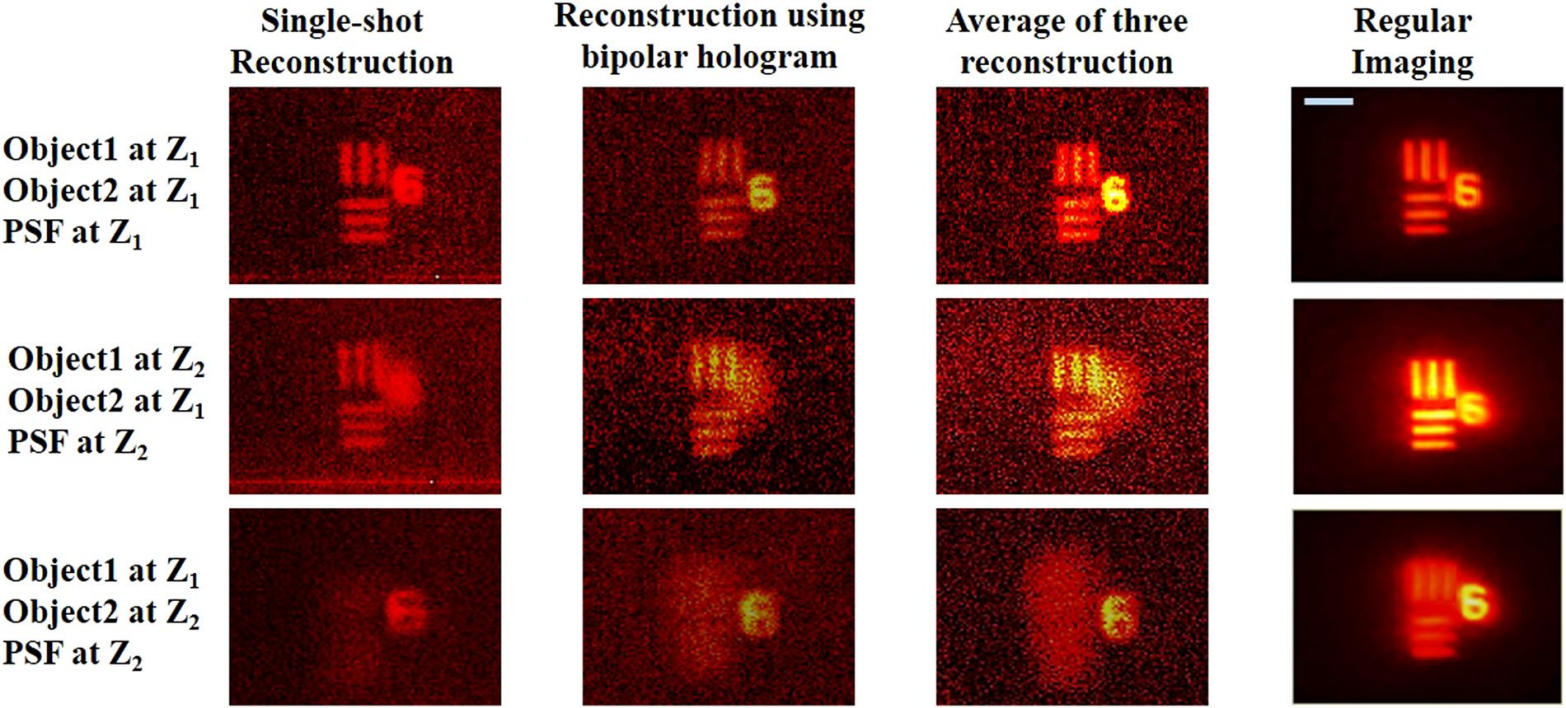
Reconstruction results of the two-plane object using PSHs corresponding to different axial locations (Δ*Z* = 3 mm). Scale bar: 350 μm.

In the next experiment, the 3D imaging capability of the imaging technique is studied. In this case, two elements in the USAF chart are considered as object 1 and object 2. Two bipolar holograms for the pinhole are synthesized using two times two intensity recordings at two different axial locations (*Z*_1_ and *Z*_2_) with a gap of Δ*Z* = 3 mm. A single bipolar object hologram is also synthesized from two intensity recordings with element 6 of group 2 and numeric digit 6 of group 2 separated by the same distance of 3 mm. The object hologram, *H*_*OBJ*_ is filtered by two filters synthesized from point object holograms *H*_*PSH1*_ and *H*_*PSH2*_, respectively. It was noted that when *H*_*OBJ*_ is filtered by the filter of *H*_*PSH1*_ the image of the object 1 was reconstructed while the image of object 2 appears blurred. If *H*_*OBJ*_ is filtered by the filter of *H*_*PSH2*_ the image of the object 2 is reconstructed while the image of the object 1 was blurred as shown in Fig. 6. The reconstruction results for a single shot, bipolar hologram without averaging and with averaging for the different cross-correlations between the object and the point object holograms are shown in Fig. 6. It can be observed that the bipolar hologram improves the SNR, and averaging three complex reconstructions further increases the SNR^35^. The reconstruction results shown in Fig. 6 confirm the 3D imaging capability of the imaging system with a scatterer. It should be noted the Fourier relation of Eq. (1) is not valid for objects outside the single plane that satisfies the imaging equation [*z*_*s*_ = *z*_*h*_*f*_1_/(*z*_*h*_ − *f*_1_)]. However, it can be shown that system properties of linearity and space invariance still exist for the other axial planes. Consequently, the reconstruction by cross-correlation with a reconstructing function calculated from the impulse response is also valid for all other axial planes.

**Figure 7:**
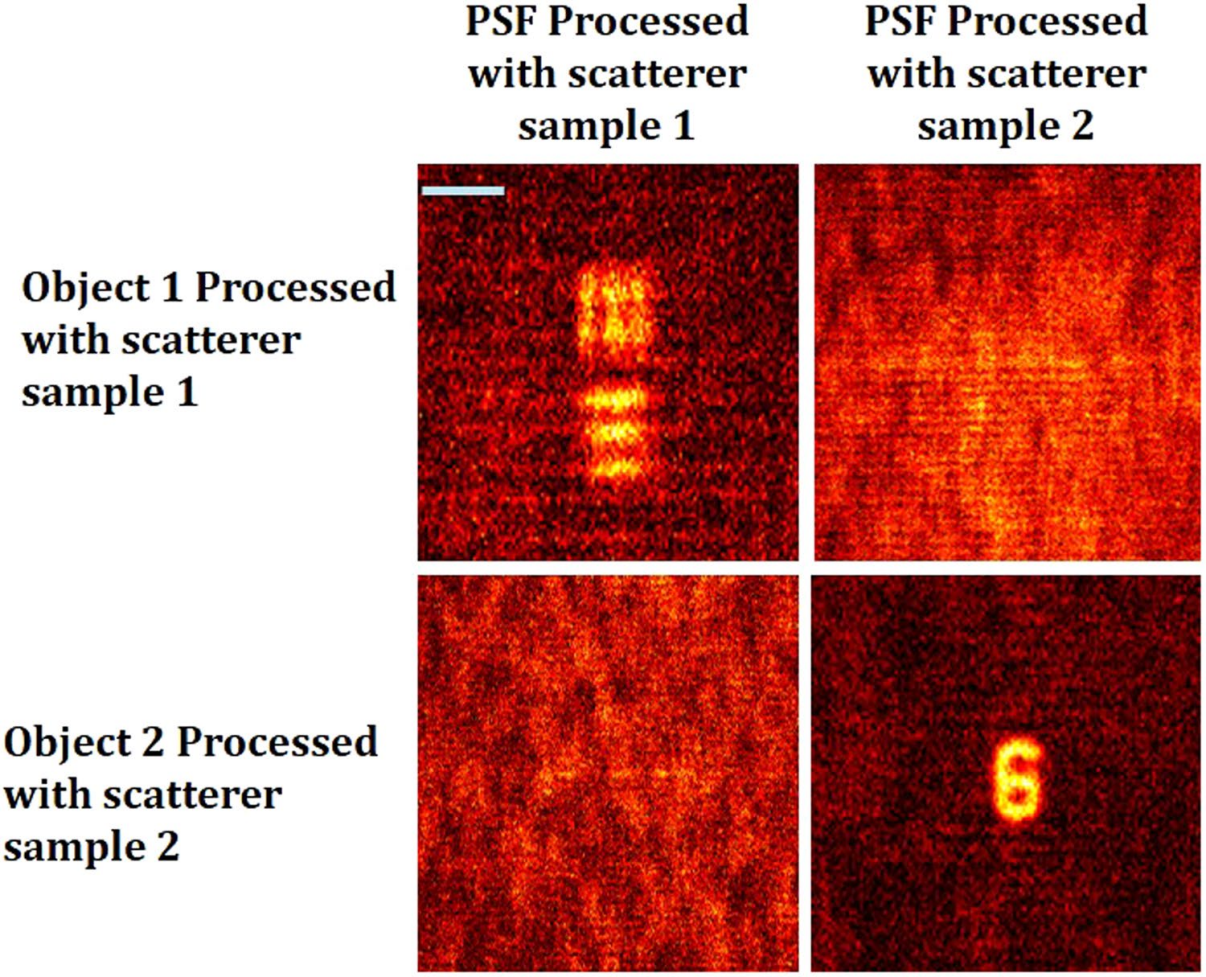
Reconstruction results using the filters of *H*_*PSH*_ (*Z*_1_, *Z*_2_) and *H*_*OBJ*_ (*Z*_1_, *Z*_2_). Scale bar: 350 μm.

We can also observe information concealment abilities of the technique i.e., only when the object hologram is reconstructed with a filter of the *H*_*PSH*_ recorded using the same scatterer and with identical experimental conditions, the image of the object was reconstructed. When a different filter was processed (using another scatterer) only a noise profile was reconstructed, as is shown in Fig. 7.

**Figure 8:**
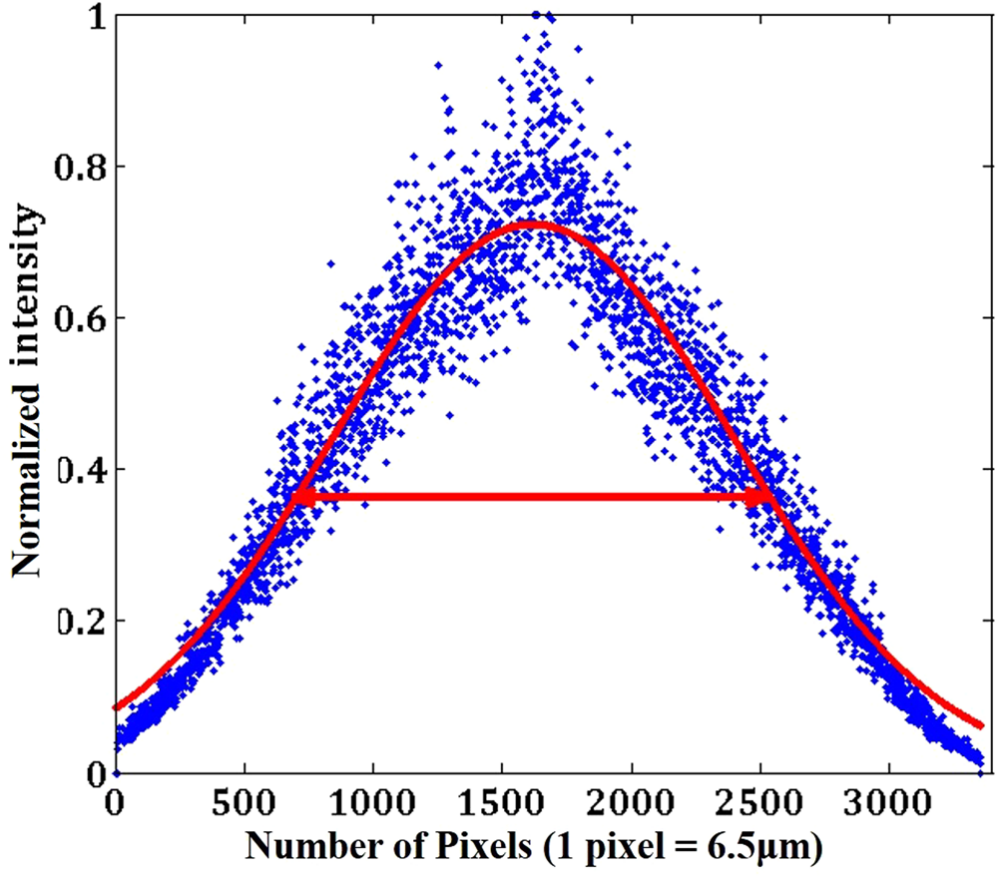
Cross-section of the spatial spectrum of the scatterer obtained using a laser with λ = 632.8 nm.

The nature of the scatterer was studied using a coherent light source (He-Ne laser) with λ = 632.8 nm. The scatterer was illuminated by a plane-wave and its spatial spectrum was recorded in the front focal plane of the lens with a focal length of 2.5 cm. The cross-section of the spatial spectrum of the scatterer is shown in Fig. 8. The FWHM of the pattern was found to be 0.752 × 10^6^ *cycles*/*m*, which means that the angle of the average scattering cone is about 26.8°.

## Conclusion

In conclusion, we have proposed and demonstrated a simple incoherent interferenceless technique to image through a scattering mask with a regular lens and a digital camera. In this technique, the scatterer is first characterized by the use of a point object and the intensity impulse response is recorded. Then, the point object is shifted to different axial locations and a library of impulse responses is created. This entire process is the training phase of the method, where it is done only once and it is suitable for imaging arbitrary number of objects. Following the training phase, an object is placed within the axial limits of the impulse response library, and another intensity pattern is recorded using the same scatterer and with identical experimental conditions. The different planes of the object are reconstructed using the different intensity impulse responses recorded at the corresponding axial locations. The experiment has been demonstrated with a single camera shot, two camera shots and also with averaging using multiple camera shots. The results with averaging showed the minimal MSE. This averaging technique can be utilized to improve the SNR by averaging over a larger number of complex reconstructions with different scatterers.

Using a point source to characterize the scatterer has some disadvantages in the sense that imaging through a scattering medium can succeed only after the medium is recognized by the system. On the other hand, the training stage of the system enables 3D imaging and can work properly with various types of scatterers. This is because the scatterer is firstly characterized and its impulse response is processed in order to optimize the imaging later.

From one perspective, the proposed technique enables imaging through a scattering medium. Therefore, this technique can be useful for seeing through natural scatterers such as human skin, tissues, fog and other turbid media. The technique uses an incoherent source and therefore can be easily utilized for fluorescence imaging and self-luminous stellar objects. From another perspective, this technique can be used to convert any regular imaging system into a 3D imager by using an easily available paper scatterer. While this technique has been demonstrated using only one lens, it can be extended to imaging systems with multiple optical components as well. The lateral resolution is limited by the 100 μm pinhole. Nonetheless, the resolution can be improved using a smaller pinhole and a powerful light source. However, it was noticed that the reconstruction was not successful for larger objects, larger than element 4 of group 2 in USAF. Therefore, additional studies are necessary to characterize the different aspects of the imaging system with a scatterer.
